# Prediction of the Physical-Mechanical Properties of Roller-Compacted Concrete Pavements under Different Service and Mix Conditions Based on Cement and Water Content

**DOI:** 10.3390/ma17030549

**Published:** 2024-01-23

**Authors:** Julián Pulecio-Díaz, Miguel Sol-Sánchez, Fernando Moreno-Navarro

**Affiliations:** 1 Faculty of Engineering, Universidad Cooperativa de Colombia, Edificio I, Ibague 730006, Colombia; 2 Laboratory of Construction Engineering, Universidad de Granada, C/Severo Ochoa s/n, 18071 Granada, Spain; msol@ugr.es (M.S.-S.); fmoreno@ugr.es (F.M.-N.)

**Keywords:** response surface methodology, water vapor per kilogram of air, physical-mechanical properties, roller-compacted concrete pavement mixes

## Abstract

Roller-compacted concrete (RCC) for pavements has experienced problems with its physical-mechanical performance over extended periods due to ambient and in situ curing conditions. Accordingly, this study aimed to present multiple regression equations for calculating the physical-mechanical properties of RCC for pavements under different service and mix conditions. For this purpose, the research included two cement and two water contents, one reduced with admixture, and four combinations of temperature and relative humidity. For model calibration and definition of the equations, cubic and beam samples were fabricated to carry out physical-mechanical tests, such as moisture content, shrinkage, and modulus of rupture. Laboratory-obtained data were studied with the Response Surface Methodology (RSM) to determine the best regression equations. The main findings determined that the behavior of a mixture of RCC at a prolonged ambient exposure time is possible because the surface models and the RSM were consistent with the different service and mix conditions. The models showed an accuracy of 98.99% in detecting shrinkage changes from 12 to 16% cement with 5.65% water in dry to wet ambient conditions. Similarly, moisture content and modulus of rupture had a 98.27 to 98.88% fit. Finally, the drying shrinkage, with mixes of 12% cement and water content variations with water-reducing admixture and superplasticizer effects, had an adjustment of 94.87%.

## 1. Introduction

Pavements have undergone significant technological advances in evaluating their performance under diverse conditions, such as varying vehicle loads, speeds, material quality, subgrade type, and specific ambient conditions. These advances include capabilities such as self-repair, recording of vehicle data (i.e., speed and load) [1], and integration with autonomous and electric vehicles. 

In addition, valuable tools have been developed for road managers, builders, and civil engineers, enabling more efficient and accurate inspections of road surfaces [2]. These tools use three-dimensional scans and semi-automated approaches to assess the condition of road surfaces both qualitatively and quantitatively [3].

On the other hand, methodologies to evaluate pavement structure by non-destructive deflection testing have been established [4]. Pavement sizing methodologies based on performance criteria have also been developed to identify the development of deterioration over time from a structural perspective and to evaluate the functional condition of pavements [5,6]. These approaches consider annual pavement-related costs, such as design, construction, maintenance, restoration, reinforcement, recycling, and reconstruction [7,8,9].

In addition, more environmentally friendly construction methods have been implemented, contributing to the sustainability of roads by reducing the generation of pollutants [10].

As a trend observed worldwide, many countries have shown interest in implementing roller-compacted concrete (RCC) pavements, adopting this category in various construction applications. Among these, ports, intermodal facilities [11,12], heavy industrial areas, light industrial areas, airport service areas, arterial streets, local streets, road widening or over-widening, berms [13], lumber facilities, composting sites, and storage yards have been highlighted as the most adequate for RCC incorporation. This preference suggests that these areas are considered vital or conducive for RCC use.

Multilayer pavement systems for high-speed roads, an important category in road construction, are not the specialty of RCC due to the need for further studies on various crucial factors. Among these, improving the texture and roughness of roller-compacted concrete pavement (RCCP) related to skid resistance and noise, among other aspects [14,15], is emphasized due to the limitations on the speed at which vehicles can circulate. This consideration favors different structures, such as flexible pavements, because they provide greater adherence to the running surface. However, they may be less resistant to static loads, low speeds [16], and extreme climates [17,18,19,20].

RCC mixtures for pavements offer different advantages, such as a more sustainable alternative [21] to traditional concrete pavements, as these require less cement [22] and can incorporate recycled materials [23,24]. These further contribute to the carbon footprint reduction policy of greater ambient sustainability [25]. However, their applicability reaches a slightly lower level of popularity than flexible pavements, as they still require further research on their resistance to cracking and their performance against short- and long-term climatic conditions [26,27].

In this context, advanced methodologies have been explored to analyze the impact of RCC pavement under varying curing conditions, primarily focusing on the relationship between temperature, compressive strength, and shrinkage [28,29,30]. Previous studies have shown that higher ambient temperatures can compromise the resistance of RCCP, leading to reduced durability and increased susceptibility to weathering effects [31,32]. This underscores the importance of understanding the behavior of this material under different temperature regimes and its implications for long-term performance.

Furthermore, field studies have revealed that diverse weather conditions, including temperature fluctuations, humidity levels, and precipitation patterns, influence shrinkage stresses. These variations in ambient conditions can lead to variable material behavior, stressing the challenge of maintaining consistent curing conditions in real-world applications [26]. As a result, it becomes crucial to assess the impact of these dynamic ambient factors on the performance of RCC pavement.

However, most published research has concentrated on assessing specific climatic scenarios, emphasizing the necessity to establish correlations between ambient conditions and key design parameters, such as cement and water content. Their goal is to develop predictive models that account for the complex interplay between these variables and the performance of the material over time. Additionally, most previously published investigations have centered on studying the response of the material after 90 days of curing submerged under water [33] to mitigate the impact of incomplete concrete maturation and its pore suction capacity [34]. This extended curing period allows for a better understanding of how RCC pavement behaves in its later development stages. It is essential to state that initial curing hours are equally significant, as this is when some of the most variable and influential phenomena may occur. Rapid changes in temperature and humidity during this early stage can substantially impact the microstructure of the material and, consequently, its long-term behavior.

State-of-the-art methodologies have revealed that RCC pavement has problems with its physical-mechanical behavior over extended periods due to the influence of the ambient and field curing conditions and when proper laboratory protocols are not applied [13,26]. This is critical in determining moisture content, shrinkage, and flexural strength.

Therefore, because of the need to correlate the parameters that govern the behavior of RCC for pavement, mathematical and statistical models have had significant contributions, especially the Response Surface Methodology (RSM) [35]. Currently, this methodology allows for the calculation of conventional mechanical properties of concretes before manufacture and material failure [36], with the addition of synthetic microfibers under freeze–thaw conditions [37], as well as the variability of physical-mechanical properties considering cement content and the water–cement ratio [38].

Accordingly, this research aims to propose multiple regression equations to calculate the physical-mechanical properties [39] of RCC pavement under different service (grams of water vapor per kilogram of air) and mix conditions (cement content [40] and water–cement ratio [26,38]) to understand the behavior of compacted pavement concrete over a long period and because it affects drying shrinkage from the beginning and after one month of manufacture.

## 2. Materials and Methods

**Figure 1:**
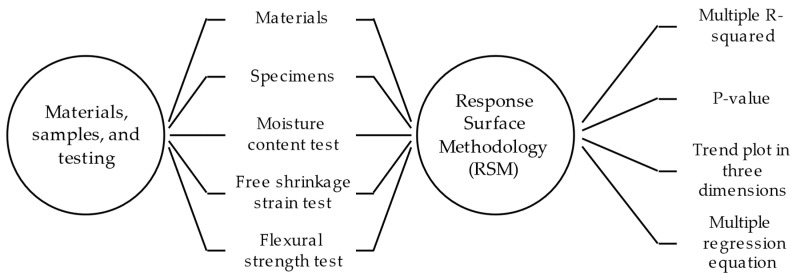
Research methodology.

**Figure 2:**
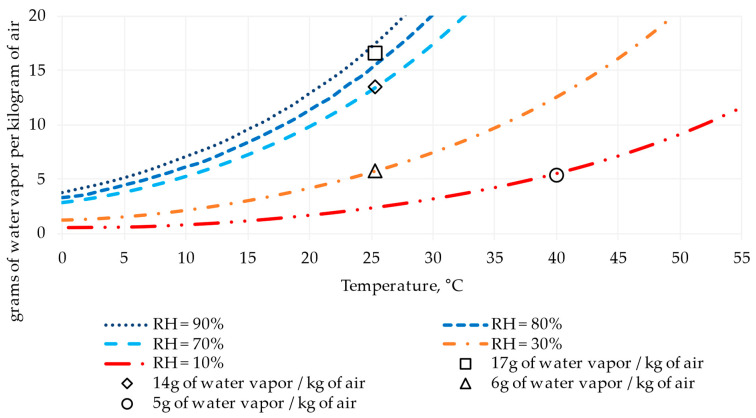
Ambient conditions, including relative humidity (RH,%), temperature (°C), and grams of water vapor per kilogram of air. Source: [42].

The methodology used in this study is comprised of two stages detailed in Figure 1: (1) materials, samples, and testing, and (2) Response Surface Methodology (RSM) [41]. The first describes the selection of materials, manufacturing of mixtures, elaboration and cutting of specimens, and laboratory tests, and the second refers to information processing utilizing the RSM inferential statistics method. Figure 2 shows the ambient conditions assessed for 90 days, which include 5, 6, 14, and 17 g of water vapor/kg of air, equivalent in this order to 85% of relative humidity (RH) at a temperature of 25 °C, 70% at 25 °C, 30% at 25 °C, and 10% at 40 °C.

### 2.1. Materials, Samples, and Testing

This study focused on three varieties of roller-compacted concrete (RCC) intended for paving. The purpose was to identify the optimal multiple regression equation, considering factors such as ambient conditions, cement, and water quantities, and using a superplasticizer that acts as a water reducer in the mix. The impacts of altering the cement ratio without changing the water content and decreasing the water content through admixtures without limiting the amount of cement were examined.

The superplasticizer MasterEase 3530 (Master Builders Solutions, Beachwood, OH, USA) was used with dosages selected following the manufacturer’s recommendations. It contained a percentage of water plus a minimum amount of the admixture, which was 0.5% of the cement weight.

**Figure 3:**
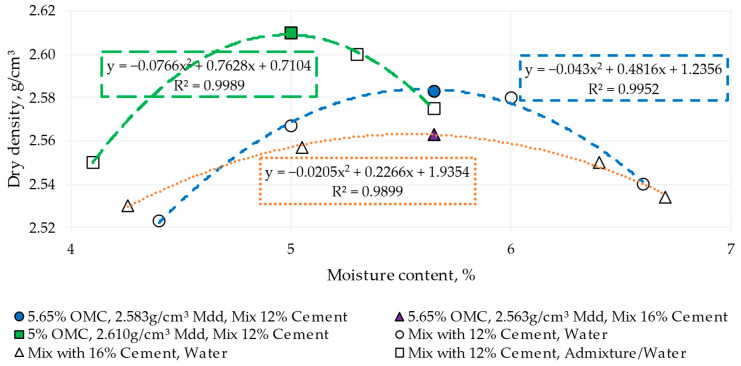
Moisture–density curves of roller-compacted cement in different mixes. OMC: optimum moisture content, Mdd: maximum dry density.

The Portland cement used in this study was of the CEM II/A-M type, composed of 80 to 88% clinker and 12 to 20% ground granulated blast furnace slag (GGBF), as well as silica fume, pozzolana, fly ash, calcined shale, limestone and up to 5% of other minor components. The cement content was determined according to the RCC literature review [43]. In contrast, the water/cement content was established using the Proctor method [21,44], which consisted of a roller-compacted concrete (RCC) specimen with a predetermined mold moisture placed in a mold in five layers. Each layer is compacted with 56 blows of a 44.48 N hammer, falling from a height of 457.2 mm, generating approximately a compaction energy of 2700 kN-m/m³. Subsequently, the dry density of the compacted sample is measured. This procedure is repeated with various molding moistures to plot a curve relating these moistures to the corresponding dry densities. This curve, known as the compaction curve, made it possible to identify the optimum moisture content (OMC) [45] of 5.65% without admixtures and 5% with admixtures and the maximum dry density for the compaction test from 2563 to 2610 g/cm^3^ (Figure 3).

**Figure 4:**
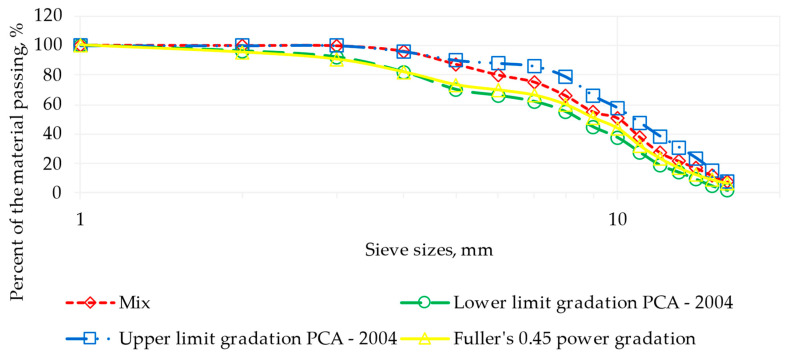
Gradation for roller-compacted concrete for pavements according to PCA [48,49].

Limestone-origin aggregates were employed with an average specific gravity [46,47] of 2.775. The specific gravities varied according to different aggregate fractions: 2.840 for 16/25, 2.805 for 10/16, 2.805 for 5.6/10, and 2.732 for 4/5.6. The total absorption was 0.472%, with individual values of 0.460, 1.020, 0.946, and 0.153% for each fraction. These aggregates correspond to the granulometric range defined by the Portland Cement Association (PCA) in 2004, as depicted in Figure 4 [48,49].

Based on the results obtained, the following parameters have been defined for each RCC mixture, detailing their composition in Table 1:

- RCC with 12% cement [43] and a 0.47 water–cement ratio (determined according to 5.65% water/12% cement).
- RCC with 16% cement and a 0.35 water–cement ratio (established according to 5.65% water/16% cement).
- RCC with 12% cement [43], an admixture of a superplasticizer with a water reducer, and a 0.42 water–cement ratio (determined according to 5.0% water/12% cement).

**Table 1:** Roller-compacted cement (RCC) mixes.

Material	W/C = 0.47 Volume (L/m^3^)	W/C = 0.35 Volume (L/m^3^)	W/C = 0.42 Volume (L/m^3^)
Cement	91.733	122.789	93.094
Oven dry weight of aggregates	-	-	-
SSD weight of aggregates	767.216	735.210	778.604
Absorbed water	-	-	-
Water at OMC	-	-	-
Free water at OMC	126.051	127.001	112.038
Admixture	-	-	1.264
1.5% air	15	15	15
Total	1000	1000	1000

SSD: saturated surface-dry, OMC: optimum moisture content, W/C: water–cement ratio.

**Figure 5:**
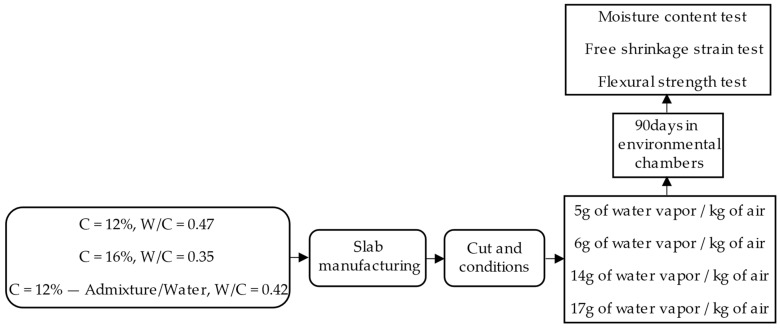
Testing plan.

Specimens were elaborated and tested according to the procedure shown in Figure 5. The following steps were carried out per specimen: (1) each mixture was manufactured using a rotary mixer; (2) molds of 100 mm × 300 mm × 300 mm were built; (3) concrete was introduced into the molds in two parts and compacted with a vibrating plate for 1 min per layer. The entire maneuver lasted 4 min; (4) when the slabs (100 mm × 300 mm × 300 mm) hardened, they were de-molded and cut with an electric diamond saw, obtaining cubes of 100 mm × 100 mm × 100 mm and beams of 100 mm × 100 mm × 285 mm; (5) the climatic chambers were prepared at 5, 6, 14, and 17 g of water vapor/kg of air where they remained for 90 days, and subsequently, moisture content, free shrinkage strain, and flexural strength tests were performed.

**Figure 6:**
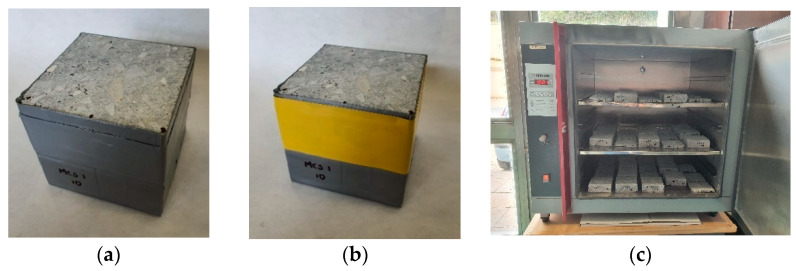
Cube samples for moisture content test. (**a**) Sealed with gray American heavy-duty adhesive tape, (**b**) the two sections of the cube sample joined with yellow tape, (**c**) samples in the oven.

Moisture content determination was performed according to the procedures described by Jafarifar et al. [33] and Asad et al. [50], which are considered to be the most modern and practical approaches in moisture measurement by gravimetric techniques. The process was carried out according to the following steps: (1) cutting the cube of 100 mm × 100 mm × 100 mm in two sections, one with a height of 10 mm and the other with a height of 90 mm; (2) the 10-mm-high section was weighed; (3) this section was sealed with gray American heavy-duty adhesive tape on four surfaces (the upper and lower surfaces were left free); (4) then, this section was weighed with the gray American heavy duty adhesive tape; (5) subsequently, the 90-mm-high section was sealed with gray American heavy-duty adhesive tape on five sides, except on the upper side, because it has to be in contact with the lower side of the 10-mm-high section; (6) the two sections of the cube were joined with yellow polyvinyl tape; (7) when all samples were ready, they were put into a climatic chamber (Universidad de Granada, Spain); (8) the samples were removed from the climatic chambers after 90 days, and the 10-mm-high section was weighed without the yellow polyvinyl tape; (9) subsequently, the 10-mm-high sections were weighed without the American gray adhesive tape; (10) then, they were placed in an oven (INDELAB, Spain) until they were dry, i.e., until achieving a constant weight; (11) finally, the moisture per day was calculated as the water–weight ratio over the dry weight. Figure 6 shows details of the procedure.

**Figure 7:**
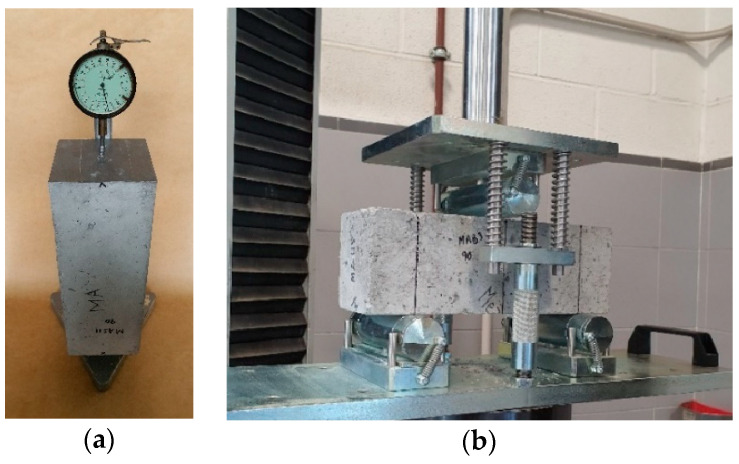
Mechanical tests. (**a**) Free shrinkage strain test, (**b**) flexural strength test.

The shrinkage tests [51] were carried out for a 90-day period, with square beam sections of 100 × 100 mm and a height of 285 mm. The procedure and calculation consisted of the following steps: (1) the beams were sealed with gray American heavy-duty adhesive tape on the lateral faces to avoid tension effects on the beam surface and internal compression [33,52], as these are usually present in the drying process; (2) blind screws on the lateral faces of each beam were installed; (3) the first measurement of the beams on the fixed frame was made with a displacement meter (ELE International, Milton Keynes, UK); (4) the beams were then placed in the climatic chambers; (5) after 90 days, the samples were taken out from the climatic chambers to be measured on the fixed frame with the same displacement meter; (6) the shrinkage strain was calculated as the height of the beam in a wet state minus the dry state over the height of the beam in a wet state using free strain measurement equipment (ELE International, Milton Keynes, UK) observed in Figure 7.

Finally, the modulus of rupture of the beams tested under shrinkage with a central point loading device and a fixed support and roller system was determined [53,54]. The test operated with a loading rate of 0.02 MPa/s, and it determined the modulus of rupture as a function of the maximum load, the distance from the support to the failure plane, and the transverse dimensions of the beams using flexural strength measurement equipment (Ibertest, Madrid, Spain) observed in Figure 7.

### 2.2. Response Surface Methodology (RSM)

The Response Surface Methodology (RSM) [55] data were analyzed with “R-4.0.5” software since it is a practical tool for research and has a great capacity to process information using advanced statistical methods. The independent and dependent variables were defined, establishing the first as the data related to cement and water vapor per kilogram of air and the second, those determined in the laboratory, included moisture content at 10 mm depth, modulus of rupture, and drying shrinkage of RCCP for 90 days.

The RSM used in the study was of first order (FO), first order with interaction (FO + TWI), and pure quadratic with interaction (PQ + TWI) unique function models. Equations (1)–(3) are the multiple regressions that are part of each model, respectively.

(1) y=b0+b1×x1+b2×x2

(2) y=b0+b1×x1+b2×x2+b12×x1×x2

(3) $$y=b0+b11\times {\left(x1\right)}^{2}+b22\times {\left(x2\right)}^{2}+b12\times x1\times x2$$

where y is the response or dependent variable, x1 and x2 are the independent variables, b0 defines the interaction of the surface with the ordinate axis, and b1, b2, b12, b11, and b22 are the partial regression coefficients. For the current study, moisture content (w10), shrinkage strain (s), and modulus of rupture (Mr) were dependent variables; x1 and x2 were the water vapor per kilogram of air (WA), cement content (C), or water–cement ratio (W/C).

**Figure 8:**
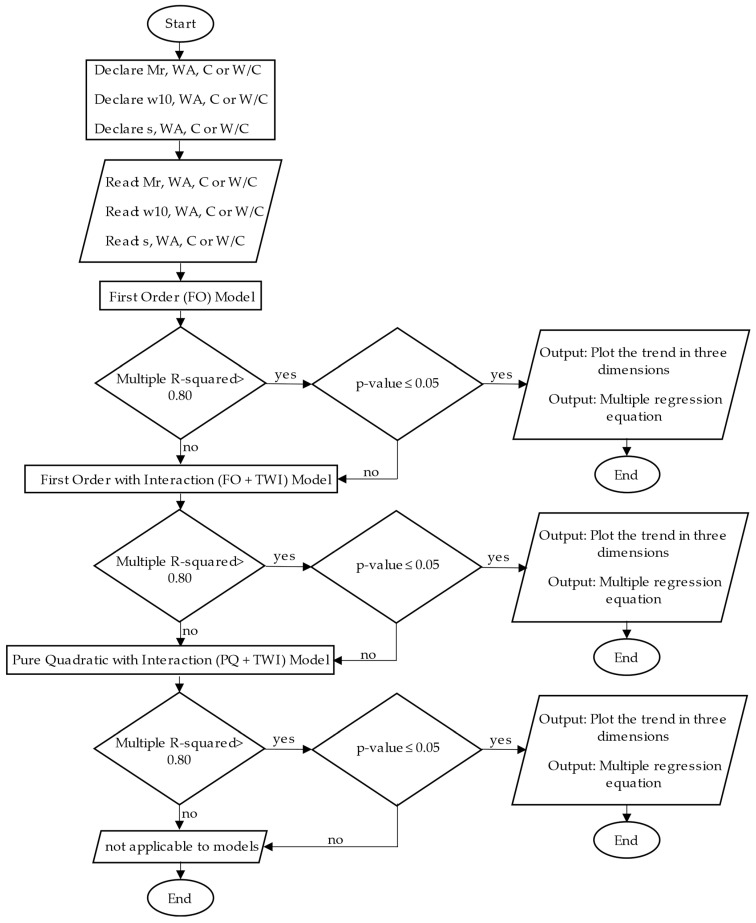
Response Surface Methodology (RSM) diagram. Mr: modulus of rupture, WA: water vapor per kilogram of air, C: cement content, W/C: water–cement ratio.

Therefore, the steps applied to use the RSM [56] are summarized in Figure 8, where a flowchart that starts, declares, and reads the input (independent) and output (dependent) data is observed. Then, a conditional procedure initiates, verifying if the multiple R-squared is higher than 0.80 and the *p*-value is less than or equal to 0.05. This process is carried out to choose the best multiple regression equation and the trend of the results through two- and three-dimensional plots. Further steps of the procedure are given below:

- Establish whether the data have a first-order, pure quadratic, or second-order behavior, considering the interaction option;
- Determine which model has the best fit. The multiple R-squared was selected as a reference point since, if the data are close to 1, it was possible to establish whether the independent variables affected the dependent ones;
- Evaluate whether the independent variables differed significantly from the dependent ones, predetermined by a *p*-value lower or equal to 0.05;
- Plot the trend in three dimensions using the Plotly function to understand the behavior of the study data.

## 3. Results and Discussion

The results were subdivided into two parts, (i) laboratory and (ii) RSM, under different service conditions (water vapor per kilogram of air) and material mix (cement, water, and admixture/water), explaining the response of the surface models and the multiple regression equations that best fit the laboratory data.

### 3.1. Laboratory Results

**Figure 9:**
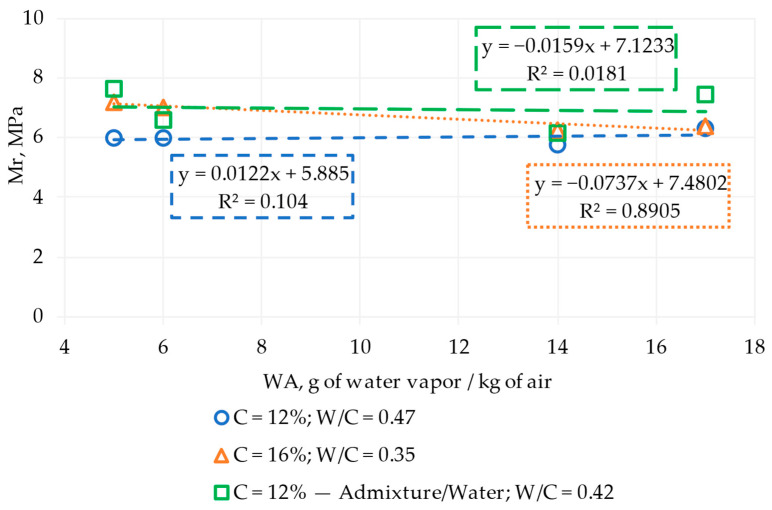
Modulus of rupture (Mr) evolution with water vapor per kilogram of air (WA) over 90 days. C: cement content, W/C: water–cement ratio.

Laboratory results in grams of water vapor per kilogram of air (WA) versus modulus of rupture in MPa (Mr) for 90 days, according to the cement content and water–cement ratio, are presented using scatter plots (Figure 9). The results evolved linearly, with growth slopes from 7 to 8% when the cement content was 16%. However, when the RCCPs were worked with 12% cement, the results showed that the graph was not linear. Therefore, the possibility of correlating the ambient and cement content with the modulus of rupture is presented since the results of the one-dimensional analysis established essential similarities. The opposite case is observed when relating the mixture of 12% cement plus admixture/water.

**Figure 10:**
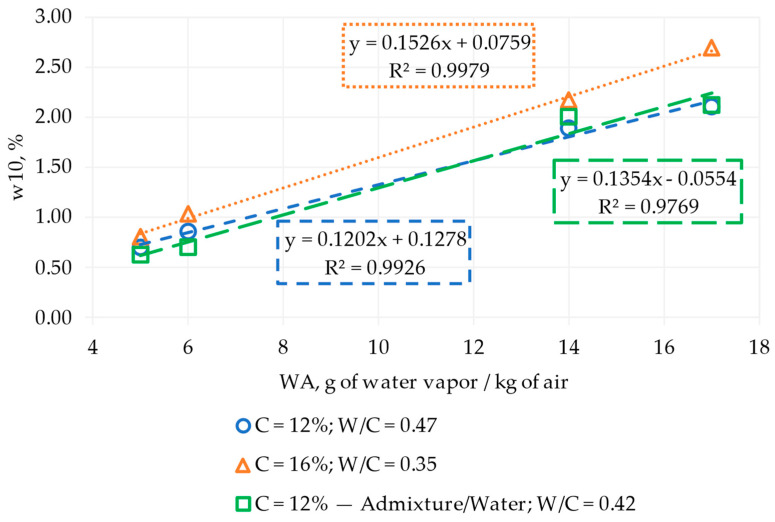
Moisture content at 10 mm depth (w10) evolution with water vapor per kilogram of air (WA) over 90 days. C: cement content, W/C: water–cement ratio.

Figure 10 shows the evolution of moisture content at a 10 mm depth (w10) with grams of water vapor per kilogram of air (WA) according to the cement content, water–cement ratio, and water reduction with admixture/water. All results evolved linearly, except for the material with admixture/water. For this material, growth slopes were found to have around 15 to 16% moisture content when the ambient conditions changed from dry to wet, and the mix worked with 16% cement and 5.65% water (W/C = 0.35). On the other hand, the mixes with 12% cement obtained growth slopes of 12% and 13 to 14% when the material was worked with 5.65% (W/C = 0.47) of water and admixture/5% of water (W/C = 0.42), respectively. The results aligned with the objectives because they established a starting point to design multiple regression equations as a function of the ambient conditions represented by grams of water vapor per kilogram of air, cement, and moisture content (see Section 3.2).

**Figure 11:**
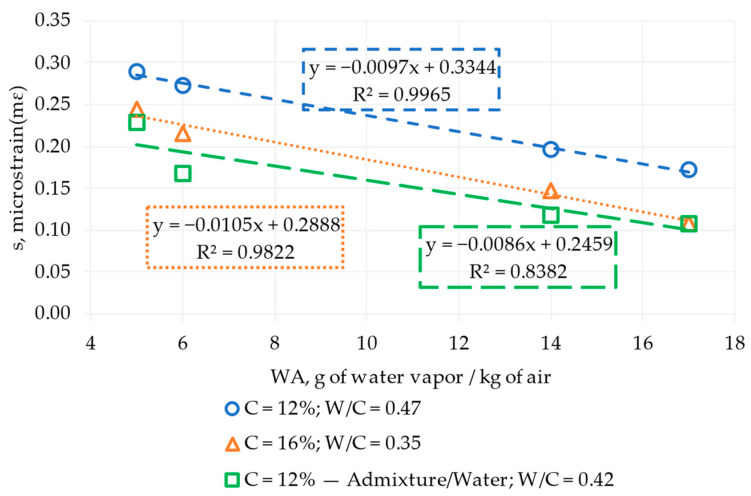
Drying shrinkage(s) evolution with water vapor per kilogram of air (WA) over 90 days. C: cement content, W/C: water–cement ratio.

Figure 11 shows the evolution of drying shrinkage versus grams of water vapor per kilogram of air as a function of cement content. The results evolved linearly, obtaining a decreasing slope of 0.97% when the RCCP had 12% cement and 5.65% water (W/C = 0.47) under dry to wet ambient conditions. Similarly, when the RCCP worked with 16% cement and 5.65% water (W/C = 0.35), a value of 1.05% was registered. In addition, when the material worked with 12% cement and admixture/5% of water (W/C = 0.42), the slope was 0.86%. The results respond to the objective because they allow for observing, from their linear behavior, a starting point from which to approach the search for multiple regression equations and, consequently, the trend of the results (see Section 3.2).

### 3.2. RSM under Different Service and Mix Conditions 

#### 3.2.1. Response Model by Cement Content

The results presented in Table 2 demonstrate that the quadratic model with interaction (PQ + TWI) is adequate to determine the modulus of rupture as a function of ambient conditions and cement content since the multiple R-squared coefficient of determination is equal to 0.9888, indicating a high correlation between the variables and the response. In addition, the *p*-value was used to validate all the variables in the model, being less than 0.05 for the sources of variation of the intercept, water vapor content squared (WA^2^), cement content squared (C^2^), and the interaction between the variables (WA, C). These results support the suitability of water vapor over air quantity and cement content as variables in the RSM model and confirm that they significantly impact the modulus of rupture under standard 90-day field curing conditions.

It is important to note that these findings agree with existing literature in this field, which has pointed out variations in material behavior and curing conditions. For example, linear behavior has been observed in studies with curing at 25 °C for 28 days [41]. This underlines the similarity between the behavior registered in this study and previous observations, further supporting the validity of the findings obtained.

**Figure 12:**
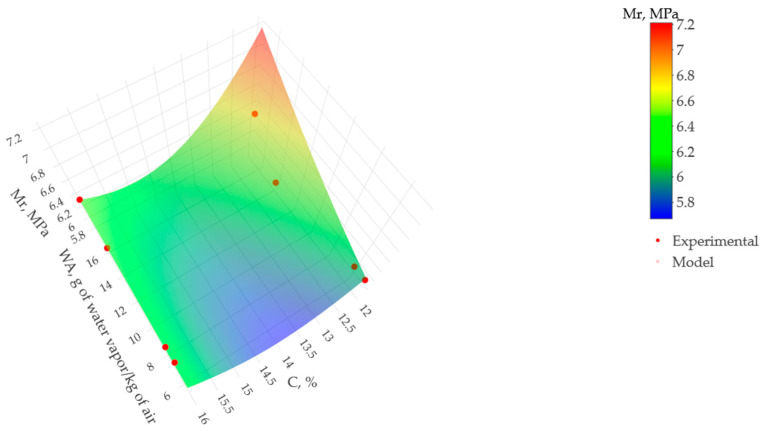
Effect of grams of water vapor per kilogram of air (WA) and cement content (C) in the modulus of rupture (Mr) over 90 days.

Once the behavior of the modulus of rupture (Mr) versus grams of water vapor per kilogram of air (WA) and cement content (C) was verified as a multiple regression equation of pure type quadratic with interaction (PQ + TWI), it was plotted. Figure 12 shows the trend of each variable, determining a higher modulus of rupture in the dry ambient, i.e., on the red surface. However, for intermediate ambient conditions located on the blue shell, lower values of the modulus of rupture are determined.

Figure 12 also shows that the modulus of rupture remains constant after 16 g of water vapor/kg of air and from 12% cement. However, from 6 to 12 g of water vapor/kg of air and 12 to 16% of cement, the modulus of rupture gradually increases. Therefore, the results determined that the RCCP blends had different trends captured by the pure quadratic with interaction (PQ + TWI) model, leading to a powerful alternative to calculating the behavior of the material after 90 days of fabrication.

The results could be instrumental in advancing the development of the multilevel back-calculation [2] approach, which implements an advanced optimization algorithm to establish pavement layer parameters accurately. This approach focuses explicitly on determining the modulus of rupture in fixed-thickness layers in roller-compacted concrete (RCC) pavements.

Table 3 presents the model fit results for moisture at a depth of 10 mm, considering the sources of intercept variation along with the variables water vapor per kilogram of air (WA) and cement content (C). In addition, *p*-values and the multiple R-squared coefficient of determination are shown for each variable and in the overall model ensemble. The model is characterized as first-order, with a multiple R-squared coefficient of determination of 0.9827, indicating a high explanatory power of the independent variables on moisture at a 10 mm depth. Furthermore, the *p*-value is less than 0.05, suggesting that the model is statistically significant. In this context, the results reveal that cement content and ambient conditions have a linear effect on moisture at this specific depth.

**Figure 13:**
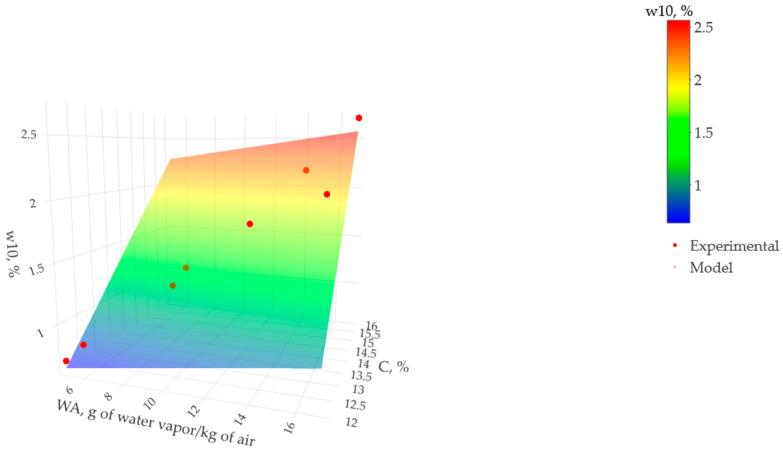
Effect of grams of water vapor per kilogram of air (WA) and cement content (C) in the moisture content at 10 mm depth (w10) over 90 days.

Figure 13 presents a flat surface model relating grams of water vapor per kilogram of air (WA), cement content (C), and moisture content (w10) in percent over 90 days. Regardless of the ambient conditions, it can be observed that from 12% cement content, the moisture content increased by 0.072% for each additional percentage unit of cement. These results indicate that the linear representation of the surface is adequate to describe the multiple regression model.

Table 4 verifies the model used to calculate drying shrinkage, considering the sources of variation as the intercept, i.e., grams of water vapor per kilogram of air (WA) and cement content (C). In addition, the *p*-values and the multiple R-squared coefficients are evaluated for each individual variable and the model as a whole, confirming the suitability of the multiple regression equation.

On this occasion, the adopted model is of first order (FO), as the multiple R-squared coefficient reaches a value of 0.9899, which is very close to 1. Furthermore, the associated *p*-value is less than 0.05, supporting the statistical significance of the model. These results indicate that the most appropriate equation for calculating drying shrinkage is a linear surface model, especially when considering the interaction between ambient conditions and cement content.

**Figure 14:**
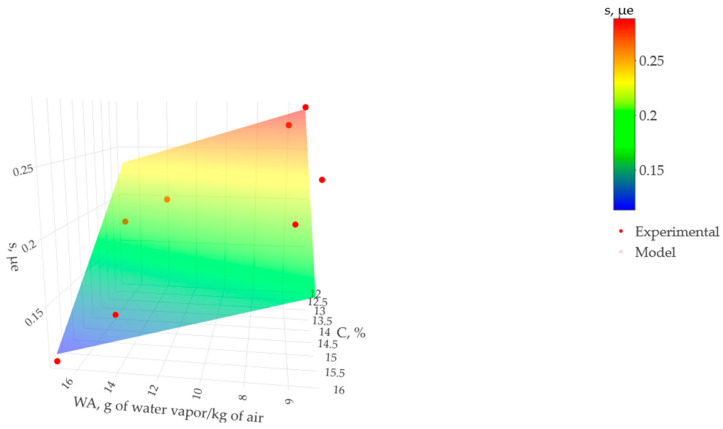
Effect of grams of water vapor per kilogram of air (WA) and cement content (C) on the drying shrinkage (s) over 90 days.

Figure 14 presents the surface model comprised of the grams of water vapor per kilogram of air (WA), the cement content in percent (C), and the drying shrinkage (s) in microstrain (mε) over 90 days. A flat and inclined surface was observed, defining cement content and drying shrinkage as linearly related. Consequently, for each ambient condition and starting at 12% cement, the shrinkage strain was calculated as decreasing by 0.013 mε (0.013 × 10^−6^) for each additional percentage unit of cement. The results determined that the linear surface adequately represents the multiple regression model.

These findings could be critical to a new version of a semi-automated pavement condition inspection tool based on three-dimensional profile scans [3]. Drying shrinkage and moisture play a crucial role in the surface microcracking of roller-compacted concrete (RCC) pavements, an essential aspect for their evaluation and maintenance.

Finally, the results showed that the surface models determined with the laboratory data were successfully validated and allowed the calculation of the physical-mechanical behavior of the RCCP mixtures at 90 days. For this purpose, Table 5 shows the multiple regression equations corresponding to each model previously studied, where each equation is a function of a mixed and a service condition variable, such as cement content in percentage (C) and grams of water vapor per kilogram of air (WA). Therefore, the results meet the main objective of the research since all the multiple regression equations meet the statistical requirements of multiple R-squared and *p*-value.

#### 3.2.2. Response Model Using the Water–Cement Ratio

Table 6 presents the results obtained using RSM. These results cover several sources of variation, including the intercept, the amount of water vapor per kilogram of air (WA), the water–cement ratio (W/C), the estimates of the constants in the multiple regression equation, the *p*-values, and the coefficient determination (R-squared).

It is important to note that the only parameter in the study that meets the fit criteria is drying shrinkage (s). The R-squared value is close to 1, and the *p*-value is less than 0.05 for each variable and the overall analysis. The results revealed that the model fits adequately to a first-degree order (FO) model.

It is relevant to underline that the study succeeded in achieving its objectives since the RCC for the pavement with a cement content of 12%, including 5.65% or an additional 5% of admixture, agrees with the multiple regression equation of a linear nature.

**Figure 15:**
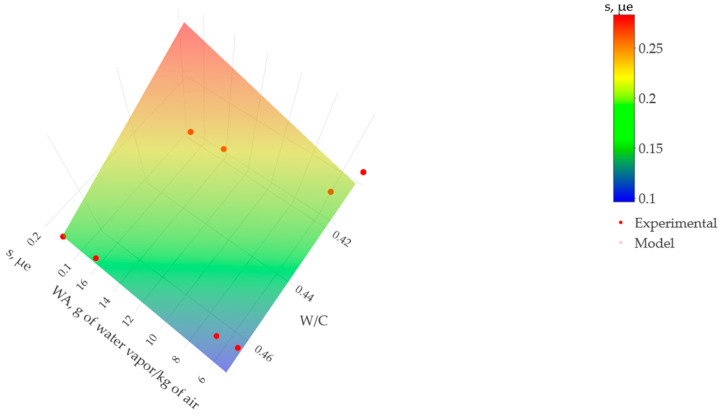
Effect of grams of water vapor per kilogram of air (WA) and water–cement ratio (W/C) on the drying shrinkage (s) over 90 days.

Figure 15 illustrates the trend observed in the relationship between grams of water vapor per kilogram of air (WA), the water–cement ratio (W/C), and drying shrinkage (s) for 90 days, considering a cement content of 12% in roller-compacted concrete. This is compared with two conditions: 5.65% water (W/C = 0.47) and an admixture representing 5% water (W/C = 0.42).

The results show an evolution under a linear trend when relating a standard amount of cement in manufacturing roller-compacted concrete, different water contents, and the inclusion of water-reducing and superplasticizing admixtures. For each ambient condition, an increase of 0.0618 mε (0.0618 × 10^−6^) in shrinkage strain is observed for every 0.04 increase in the water–cement ratio. These findings indicate that the linear surface adequately represents the underlying multiple regression model.

Finally, the surface models calculated with the water–cement ratio and the grams of water vapor per kilogram of air (WA) were satisfactorily validated and allowed for calculating over 90 days the drying shrinkage (s) behavior of the RCCP mixes under a standard cement content of 12% and various amounts of water related to a water-reducing admixture and a superplasticizer. For this purpose, Table 7 shows the equations with their respective regression constants, multiple R-squared, *p*-value, and model. Specifically, the results meet the main objective of the research since the regression equation met the statistical requirements of the R-squared and *p*-value. 

## 4. Conclusions

The current research created multiple regression equations for a 90-day calculation period of the physical-mechanical properties of roller-compacted concrete (RCC) for pavement under normal ambient conditions, such as the amount of water vapor per kilogram of air, evaluating the development of moisture content, shrinkage strain, and flexural strength. In addition, the materials studied had variations in the amount of cement and water in the manufacturing process. From the results obtained, the following conclusions are drawn:

- The laboratory results fit the multiple regression equations because they approach one in the R-squared, and the *p*-value of the statistical test is less than 0.05. These findings determined a pure quadratic model with interaction (PQ + TWI) for the modulus of rupture, similar to what was found in state-of-the-art studies, and a first-order (FO) model for drying shrinkage and moisture content.
- The study indicates that the modulus of rupture of roller-compacted concrete varies with changes in ambient conditions and cement content. With 12% cement, the strength decreases by 4% in ambient conditions with 6–10 g of water vapor per kilogram of air but increases by 4.2% with 10–16 g of vapor. Increasing the cement to 16% reduces the strength to 8% in ambient conditions of 6–14 g of vapor but only 1% in 14–16 g. It indicates that the relationship between strength and ambient conditions is not linear, suggesting using non-linear calculation models for accurate estimations.
- In roller-compacted concrete (RCC) mixes with a cement content of 12%, drying shrinkage has been registered to decrease by up to 14% with each increase of 3 to 4 g of water vapor per kilogram of air. This reduction is even more significant, reaching 18%, when the proportion of cement increases to 16%. This finding is especially relevant when contrasted with the behavior of moisture content since mixtures with a higher percentage of cement tend to absorb more water from the ambient conditions. Therefore, it is concluded that raising the cement content from 12% to 16% in RCC mixes intended for paving leads to an additional decrease of up to 4% in drying shrinkage for each increase of 3 to 4 g of water vapor per kilogram of air.
- The multiple regression equation for calculating drying shrinkage in mixes of 12% cement, 5.65% (W/C = 0.47) water, and admixture/5% water (W/C = 0.42) reveals that shrinkage decreases by 49% with each increase of 3 to 4 g of water vapor per kilogram of air when the water–cement ratio is 0.36. However, in mixtures with higher water content (water–cement ratio of 0.48), the reduction in shrinkage can be as high as 12%. It emphasizes the importance of developing a regression equation that considers mixes with identical cement content but varies the amount of water and admixtures.
- Finally, there is the possibility to expand the research to analyze the physical-mechanical properties of RCC mixes under service conditions with other admixtures in everyday practice. Likewise, the integration of sustainable materials, such as alternative aggregates derived from recycled concrete, recycled asphalt aggregates, electric arc furnaces, metallurgical slag, and recycled tire materials, such as rubber granules and polypropylene fibers, should be considered.

## Figures and Tables

**Table 2:** Effect of the water vapor per kilogram of air (WA) and cement content (C) in the modulus of rupture (Mr) over 90 days using the Response Surface Methodology.

Source of Variation	Estimate	Std. Error	t Value	*p*-Value	Significant Difference (Only *p*-Values ≤ 0.05 Are Significant)
Intercept	4.9462376	0.1016573	48.66	1.07 × 10^−6^	Yes
Pure quadratic (WA, C) WA^2^	0.0130429	0.0013011	10.02	0.000557	Yes
Pure quadratic (WA, C) C^2^	0.0145394	0.0008686	16.74	7.46 × 10^−5^	Yes
Two-way interaction (WA, C)	−0.0222764	0.0019941	−11.17	0.000366	Yes

Model: pure quadratic with interaction (PQ + TWI); multiple R-squared: 0.9888; *p*-value: 0.0002337.

**Table 3:** Effect of the water vapor per kilogram of air (WA) and cement content (C) in the moisture content at 10 mm depth (w10) over 90 days using the Response Surface Methodology.

Source of Variation	Estimate	Std. Error	t Value	*p*-Value	Significant Difference (Only *p*-Values ≤ 0.05 Are Significant)
Intercept	−0.9069172	0.3113942	−2.9124	0.03331	Yes
WA	0.1363643	0.0082556	16.5179	1.485 × 10^−5^	Yes
C	0.0720525	0.0211486	3.4070	0.01911	Yes

Model: first-order (FO); multiple R-squared: 0.9827; *p*-value: 3.922 × 10^−5^.

**Table 4:** Effect of the water vapor per kilogram of air (WA) and cement content (C) on the drying shrinkage (s) at 90 days using the Response Surface Methodology.

Source of Variation	Estimate	Std. Error	t Value	*p*-Value	Significant Difference (Only *p*-Values ≤ 0.05 Are Significant)
Intercept	0.50085773	0.01933573	25.903	1.602 × 10^−6^	Yes
WA	−0.01006363	0.00051262	−19.632	6.332 × 10^−6^	Yes
C	−0.01351872	0.00131320	−10.294	0.0001487	Yes

Model: first-order (FO); multiple R-squared: 0.9899; *p*-value: 1.018 × 10^−5^.

**Table 5:** Multiple regression equations under different service and cement content (C) conditions.

Equation	Regression Constants	Multiple R-Squared	*p*-Value	Model
$Mr=b0+b11\times {\left(WA\right)}^{2}+b22\times {\left(C\right)}^{2}+b12\times WA\times C$	b0 = 4.9462376	0.9888	0.0002337	PQ + TWI
	b11 = 0.0130429			
	b22 = 0.0145394			
	b12 = −0.0222764			
w10=b0+b1×WA+b2×C	b0 = −0.9069172	0.9827	3.922 × 10^−5^	FO
	b1 = 0.1363643			
	b2 = 0.0720525			
s=b0+b1×WA+b2×C	b0 = 0.50085773	0.9899	1.018 × 10^−5^	FO
	b1 = −0.01006363			
	b2 = −0.01351872			

Mr: modulus of rupture, WA: water vapor per kilogram of air, w10: moisture content at 10 mm depth, s: drying shrinkage, C: cement content, PQ + TWI: pure quadratic with interaction, FO: first order.

**Table 6:** Effect of the water vapor per kilogram of air (WA) and water–cement ratio (W/C) on the drying shrinkage (s) over 90 days using the response surface methodology.

Source of Variation	Estimate	Std. Error	t Value	*p*-Value	Significant Difference (Only *p*-Values ≤ 0.05 Are Significant)
Intercept	−0.3977277	0.1131583	−3.5148	0.0170134	Yes
WA	−0.0091270	0.0012307	−7.4159	0.0007021	Yes
W/C	1.5458007	0.2522270	6.1286	0.0016786	Yes

Model: first-order (FO); multiple R-squared: 0.9487; *p*-value: 0.0005947.

**Table 7:** Multiple regression equations under different service and water–cement ratio (W/C) conditions.

Equation	Regression Constants	Multiple R-Squared	*p*-Value	Model
s=b0+b1×WA+b2×(W/C)	b0 = −0.3977277	0.9487	0.0005947	FO
	b1 = −0.0091270			
	b2 = 1.5458007			

s: drying shrinkage, WA: water vapor per kilogram of air, W/C: water–cement ratio, FO: first-order model.

## Data Availability

Data are contained within the article.

## Abbreviations

The following abbreviations are used in this manuscript:

C	Cement content
CEM II/A-M	Portland Composite Cement
FO	First Order
FO + TWI	First Order with Interaction
PQ + TWI	Pure Quadratic with Interaction
GGBF	Ground Granulated Blast Furnace
Mr	Modulus of Rupture
OMC	Optimum Moisture Content
RCC	Roller-Compacted Concrete
RCCP	Roller Compacted Concrete for Pavements
RH	Relative Humidity
RSM	Response Surface Methodology
s	Shrinkage Strain
T	Temperature
WA	Water Vapor per Kilogram of Air
W/C	Water–Cement Ratio
w10	Moisture Content
